# 2-(3-Chloro­phen­yl)-4,5-dihydro-1*H*-imidazole

**DOI:** 10.1107/S1600536809001214

**Published:** 2009-01-17

**Authors:** Reza Kia, Hoong-Kun Fun, Hadi Kargar

**Affiliations:** a X-ray Crystallography Unit, School of Physics, Universiti Sains Malaysia, 11800 USM, Penang, Malaysia; b Department of Chemistry, School of Science, Payame Noor University (PNU), Ardakan, Yazd, Iran

## Abstract

In the title compound, C_9_H_9_ClN_2_, a substituted imidazoline, the six- and five-membered rings are twisted from each other, making a dihedral angle of 17.07 (5)°. In the crystal structure, a short Cl⋯Cl [3.3540 (3) Å] inter­action is observed. Neighbouring mol­ecules are linked together by inter­molecular N—H⋯N hydrogen bonds into a one-dimensional infinite chain along the [101] direction and short Cl⋯Cl contacts link the chains into a three-dimensional network. There is also a significant π-stacking inter­action between the planar sections of the six- and five-membered rings.

## Related literature

For bond-length data, see: Allen *et al.* (1987 ▶). For a related structure and the synthesis, see: Stibrany *et al.* (2004 ▶); Kia *et al.* (2008 ▶). For the biological and pharmacological activities of imidazoline derivatives, see, for example: Blancafort (1978 ▶); Chan (1993 ▶); Vizi (1986 ▶); Li *et al.* (1996 ▶); Ueno *et al.* (1995 ▶); Corey & Grogan (1999 ▶).
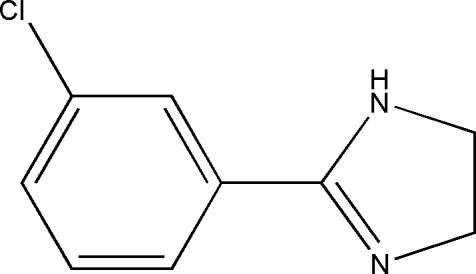


## Experimental

### 

#### Crystal data

C_9_H_9_ClN_2_
*M* *_r_* = 180.63
Orthorhombic, 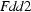

*a* = 19.7329 (8) Å
*b* = 39.1479 (18) Å
*c* = 4.3493 (2) Å
*V* = 3359.8 (3) Å^3^
*Z* = 16
Mo *K*α radiation
μ = 0.39 mm^−1^
*T* = 100.0 (1) K
0.51 × 0.50 × 0.09 mm

#### Data collection

Bruker APEXII CCD area-detector diffractometer
Absorption correction: multi-scan (**SADABS**; Bruker, 2005 ▶) *T*_min_ = 0.825, *T*_max_ = 0.964
14166 measured reflections
3438 independent reflections
3224 reflections with *I* > 2σ(*I*)
*R*_int_ = 0.025

#### Refinement

*R*[*F* ^2^ > 2σ(*F* ^2^)] = 0.027
*wR*(*F* ^2^) = 0.072
*S* = 1.10
3438 reflections
113 parameters
1 restraint
H atoms treated by a mixture of independent and constrained refinement
Δρ_max_ = 0.33 e Å^−3^
Δρ_min_ = −0.15 e Å^−3^
Absolute structure: Flack (1983 ▶), 1429 Friedel pairs
Flack parameter: −0.05 (4)

### 

Data collection: *APEX2* (Bruker, 2005 ▶); cell refinement: *SAINT* (Bruker, 2005 ▶); data reduction: *SAINT*; program(s) used to solve structure: *SHELXTL* (Sheldrick, 2008 ▶); program(s) used to refine structure: *SHELXTL*; molecular graphics: *SHELXTL*; software used to prepare material for publication: *SHELXTL* and *PLATON* (Spek, 2003 ▶).

## Supplementary Material

Crystal structure: contains datablocks global, I. DOI: 10.1107/S1600536809001214/is2379sup1.cif

Structure factors: contains datablocks I. DOI: 10.1107/S1600536809001214/is2379Isup2.hkl

Additional supplementary materials:  crystallographic information; 3D view; checkCIF report

## Figures and Tables

**Table 1:** Selected interatomic distances (Å)

Cl1⋯Cl1^i^	3.3540 (3)
C1⋯C3^ii^	3.3945 (12)
C1⋯C4^ii^	3.3301 (15)
C4⋯C6^iii^	3.3997 (15)
C5⋯C7^iii^	3.3716 (12)

Symmetry codes: (i) 
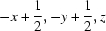
; (ii) 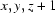; (iii) 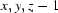.

**Table 2:** Hydrogen-bond geometry (Å, °)

*D*—H⋯*A*	*D*—H	H⋯*A*	*D*⋯*A*	*D*—H⋯*A*
N1—H1*N*1⋯N2^iv^	0.896 (16)	2.118 (16)	3.0113 (11)	174.5 (15)

Symmetry code: (iv) 
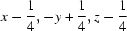
.

**Fig. 1.**
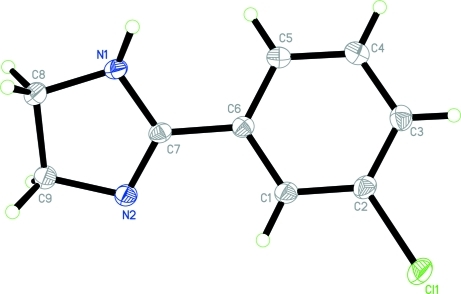
The molecular structure of (I) with atom labels and 50% probability ellipsoids for non-H atoms.

**Fig. 2.**
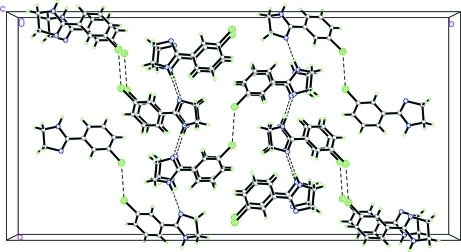
The crystal packing of (I), viewed down the c-axis showing linking of molecules through intermolecular N—H···N hydrogen bonds and short Cl···Cl interactions. The intermolecular interactions are shown as dashed lines.

## supplementary crystallographic information

### Comment

Imidazoline derivatives are of great importance because they exhibit significant biological and pharmacological activities including antihypertensive (Blancafort, 1978), antihyperglycemic (Chan, 1993), antidepressive (Vizi, 1986), antihypercholesterolemic (Li *et al.,* 1996) and antiinflammatory (Ueno *et al.,* 1995) properties. These compounds are also used as catalysts and synthetic intermediates in some organic reactions (Corey & Grogan, 1999). Due to these important applications of imidazolines, here we report the crystal structure of the title compound, (I).

In the title compound (Fig. 1), bond lengths (Allen *et al.*, 1987) and angles are within the normal ranges and are comparable with the related structures (Stibrany *et al.*, 2004; Kia *et al.,* 2008). The six- and five-membered rings are not coplanar and are twisted from each other by a dihedral angle of 18.07 (5)°. The interesting feature of the crystal structure is the short Cl···Cl [3.3540 (3) Å] (Table 1) which is shorter than the sum of the van der Waals radius of this atom. In the crystal structure (Fig. 2), neighbouring molecules are linked together by intermolecular N—H···N hydrogen bonds (Table 2) into 1-D infinite chains along the [1 0 1] direction and short Cl···Cl contacts link these chains into a 3-D network. There is also a significant π-stacking interaction between the planar sections associated with C1–C3–C4–C5–C6 and C7 of the six- and five-membered rings respectively (Table 1).

### Experimental

The synthetic method was based on the previous work (Stibrany *et al.*, 2004), except that 10 mmol of 3-chloro-2-cyanobenzene and 40 mmol of ethylenediamine were used. Single crystals suitable for *X*-ray diffraction were obtained by evaporation of an acetonitrile solution at room temperature.

### Refinement

The H atom bound to N1 was located in a difference Fourier map and refined freely. Other H atoms were positioned geometrically and refined in a riding model approximation, with C—H = 0.93–0.97 Å, and with *U*_iso_(H) = 1.2*U*_eq_(C).

### Crystal data

**Table:**

C_9_H_9_ClN_2_	*F*(000) = 1504
*M**_r_* = 180.63	*D*_x_ = 1.428 Mg m^−^^3^
Orthorhombic, *F**d**d*2	Mo *K*α radiation, λ = 0.71073 Å
Hall symbol: F 2 -2d	Cell parameters from 8962 reflections
*a* = 19.7329 (8) Å	θ = 2.9–36.7°
*b* = 39.1479 (18) Å	µ = 0.39 mm^−^^1^
*c* = 4.3493 (2) Å	*T* = 100 K
*V* = 3359.8 (3) Å^3^	Block, colourless
*Z* = 16	0.51 × 0.50 × 0.09 mm

### Data collection

**Table:**

Bruker APEXII CCD area-detector diffractometer	3438 independent reflections
Radiation source: fine-focus sealed tube	3224 reflections with *I* > 2σ(*I*)
graphite	*R*_int_ = 0.025
φ and ω scans	θ_max_ = 35.0°, θ_min_ = 2.1°
Absorption correction: multi-scan (*SADABS*; Bruker, 2005)	*h* = −31→25
*T*_min_ = 0.825, *T*_max_ = 0.964	*k* = −60→60
14166 measured reflections	*l* = −6→6

### Refinement

**Table:**

Refinement on *F*^2^	Secondary atom site location: difference Fourier map
Least-squares matrix: full	Hydrogen site location: inferred from neighbouring sites
*R*[*F*^2^ > 2σ(*F*^2^)] = 0.027	H atoms treated by a mixture of independent and constrained refinement
*wR*(*F*^2^) = 0.072	*w* = 1/[σ^2^(*F*_o_^2^) + (0.037*P*)^2^ + 1.1492*P*] where *P* = (*F*_o_^2^ + 2*F*_c_^2^)/3
*S* = 1.10	(Δ/σ)_max_ = 0.001
3438 reflections	Δρ_max_ = 0.33 e Å^−^^3^
113 parameters	Δρ_min_ = −0.15 e Å^−^^3^
1 restraint	Absolute structure: Flack (1983), 1429 Friedel pairs
Primary atom site location: structure-invariant direct methods	Flack parameter: −0.05 (4)

### Special details

**Table:**

Experimental. The low-temperature data was collected with the Oxford Cyrosystem Cobra low-temperature attachment.
Geometry. All e.s.d.'s (except the e.s.d. in the dihedral angle between two l.s. planes) are estimated using the full covariance matrix. The cell e.s.d.'s are taken into account individually in the estimation of e.s.d.'s in distances, angles and torsion angles; correlations between e.s.d.'s in cell parameters are only used when they are defined by crystal symmetry. An approximate (isotropic) treatment of cell e.s.d.'s is used for estimating e.s.d.'s involving l.s. planes.
Refinement. Refinement of *F*^2^ against ALL reflections. The weighted *R*-factor *wR* and goodness of fit *S* are based on *F*^2^, conventional *R*-factors *R* are based on *F*, with *F* set to zero for negative *F*^2^. The threshold expression of *F*^2^ > σ(*F*^2^) is used only for calculating *R*-factors(gt) *etc*. and is not relevant to the choice of reflections for refinement. *R*-factors based on *F*^2^ are statistically about twice as large as those based on *F*, and *R*- factors based on ALL data will be even larger.

### Fractional atomic coordinates and isotropic or equivalent isotropic displacement parameters (Å^2^)

**Table:**

	*x*	*y*	*z*	*U*_iso_*/*U*_eq_	
Cl1	0.165233 (12)	0.246951 (6)	0.02330 (7)	0.02846 (7)	
N1	0.00209 (4)	0.10678 (2)	0.1715 (2)	0.01782 (15)	
N2	0.11053 (4)	0.11676 (2)	0.3205 (2)	0.01792 (15)	
C1	0.10570 (4)	0.18532 (2)	0.0803 (2)	0.01732 (16)	
H1A	0.1405	0.1803	0.2172	0.021*	
C2	0.10314 (4)	0.21685 (2)	−0.0634 (2)	0.01837 (17)	
C3	0.05234 (5)	0.22529 (2)	−0.2708 (2)	0.01901 (17)	
H3A	0.0517	0.2465	−0.3667	0.023*	
C4	0.00232 (5)	0.20104 (3)	−0.3313 (3)	0.01959 (17)	
H4A	−0.0323	0.2062	−0.4686	0.024*	
C5	0.00358 (5)	0.16926 (2)	−0.1890 (2)	0.01739 (16)	
H5A	−0.0301	0.1533	−0.2313	0.021*	
C6	0.05544 (4)	0.16118 (2)	0.0174 (2)	0.01498 (14)	
C7	0.05755 (4)	0.12791 (2)	0.1766 (2)	0.01505 (15)	
C8	0.01553 (5)	0.07848 (3)	0.3841 (3)	0.02052 (18)	
H8A	0.0053	0.0566	0.2907	0.025*	
H8B	−0.0102	0.0809	0.5728	0.025*	
C9	0.09225 (5)	0.08268 (3)	0.4421 (3)	0.02013 (18)	
H9A	0.1021	0.0813	0.6602	0.024*	
H9B	0.1175	0.0650	0.3364	0.024*	
H1N1	−0.0392 (8)	0.1159 (4)	0.148 (4)	0.036 (4)*	

### Atomic displacement parameters (Å^2^)

**Table:**

	*U*^11^	*U*^22^	*U*^33^	*U*^12^	*U*^13^	*U*^23^
Cl1	0.01974 (10)	0.01899 (11)	0.04665 (16)	−0.00554 (8)	−0.00483 (11)	0.00392 (11)
N1	0.0124 (3)	0.0167 (4)	0.0244 (4)	−0.0016 (3)	−0.0022 (3)	0.0014 (3)
N2	0.0132 (3)	0.0161 (3)	0.0245 (4)	0.0007 (3)	−0.0013 (3)	0.0019 (3)
C1	0.0122 (3)	0.0165 (4)	0.0232 (4)	0.0008 (3)	−0.0002 (3)	−0.0003 (3)
C2	0.0139 (3)	0.0166 (4)	0.0246 (4)	−0.0010 (3)	0.0019 (3)	−0.0004 (3)
C3	0.0189 (4)	0.0171 (4)	0.0210 (4)	0.0013 (3)	0.0025 (3)	0.0013 (3)
C4	0.0180 (4)	0.0211 (4)	0.0196 (4)	0.0016 (3)	−0.0018 (3)	0.0000 (3)
C5	0.0148 (3)	0.0192 (4)	0.0181 (4)	0.0000 (3)	−0.0006 (3)	−0.0007 (3)
C6	0.0120 (3)	0.0149 (4)	0.0180 (4)	0.0012 (3)	0.0018 (3)	−0.0014 (3)
C7	0.0117 (3)	0.0158 (4)	0.0177 (4)	−0.0003 (3)	0.0011 (3)	−0.0016 (3)
C8	0.0164 (4)	0.0188 (4)	0.0264 (4)	−0.0021 (3)	−0.0011 (3)	0.0042 (3)
C9	0.0159 (4)	0.0184 (4)	0.0260 (5)	0.0002 (3)	−0.0008 (3)	0.0039 (3)

### Geometric parameters (Å, °)

**Table:**

Cl1—C2	1.7413 (10)	C3—H3A	0.9300
N1—C7	1.3719 (12)	C4—C5	1.3897 (14)
N1—C8	1.4675 (13)	C4—H4A	0.9300
N1—H1N1	0.896 (16)	C5—C6	1.3976 (13)
N2—C7	1.2942 (12)	C5—H5A	0.9300
N2—C9	1.4799 (13)	C6—C7	1.4759 (13)
C1—C2	1.3844 (14)	C8—C9	1.5436 (13)
C1—C6	1.3969 (12)	C8—H8A	0.9700
C1—H1A	0.9300	C8—H8B	0.9700
C2—C3	1.3884 (14)	C9—H9A	0.9700
C3—C4	1.3944 (14)	C9—H9B	0.9700
			
Cl1···Cl1^i^	3.3540 (3)	C4···C6^iii^	3.3997 (15)
C1···C3^ii^	3.3945 (12)	C5···C7^iii^	3.3716 (12)
C1···C4^ii^	3.3301 (15)		
			
C7—N1—C8	107.50 (7)	C1—C6—C5	119.51 (8)
C7—N1—H1N1	119.1 (10)	C1—C6—C7	119.03 (8)
C8—N1—H1N1	122.5 (11)	C5—C6—C7	121.45 (8)
C7—N2—C9	106.25 (7)	N2—C7—N1	116.68 (8)
C2—C1—C6	119.26 (9)	N2—C7—C6	123.17 (8)
C2—C1—H1A	120.4	N1—C7—C6	120.13 (8)
C6—C1—H1A	120.4	N1—C8—C9	101.53 (7)
C1—C2—C3	122.13 (9)	N1—C8—H8A	111.5
C1—C2—Cl1	118.68 (7)	C9—C8—H8A	111.5
C3—C2—Cl1	119.19 (8)	N1—C8—H8B	111.5
C2—C3—C4	118.15 (9)	C9—C8—H8B	111.5
C2—C3—H3A	120.9	H8A—C8—H8B	109.3
C4—C3—H3A	120.9	N2—C9—C8	106.06 (8)
C5—C4—C3	120.84 (9)	N2—C9—H9A	110.5
C5—C4—H4A	119.6	C8—C9—H9A	110.5
C3—C4—H4A	119.6	N2—C9—H9B	110.5
C4—C5—C6	120.12 (8)	C8—C9—H9B	110.5
C4—C5—H5A	119.9	H9A—C9—H9B	108.7
C6—C5—H5A	119.9		
			
C6—C1—C2—C3	−0.48 (14)	C9—N2—C7—C6	−179.11 (8)
C6—C1—C2—Cl1	178.76 (7)	C8—N1—C7—N2	9.55 (12)
C1—C2—C3—C4	0.66 (14)	C8—N1—C7—C6	−171.73 (8)
Cl1—C2—C3—C4	−178.58 (8)	C1—C6—C7—N2	−15.38 (13)
C2—C3—C4—C5	−0.37 (15)	C5—C6—C7—N2	166.02 (9)
C3—C4—C5—C6	−0.09 (15)	C1—C6—C7—N1	165.98 (9)
C2—C1—C6—C5	0.00 (13)	C5—C6—C7—N1	−12.62 (13)
C2—C1—C6—C7	−178.62 (8)	C7—N1—C8—C9	−13.33 (10)
C4—C5—C6—C1	0.28 (14)	C7—N2—C9—C8	−8.35 (11)
C4—C5—C6—C7	178.87 (9)	N1—C8—C9—N2	13.08 (10)
C9—N2—C7—N1	−0.43 (11)		

Symmetry codes: (i) −*x*+1/2, −*y*+1/2, *z*; (ii) *x*, *y*, *z*+1; (iii) *x*, *y*, *z*−1.

### Hydrogen-bond geometry (Å, °)

**Table:**

*D*—H···*A*	*D*—H	H···*A*	*D*···*A*	*D*—H···*A*
N1—H1N1···N2^iv^	0.896 (16)	2.118 (16)	3.0113 (11)	174.5 (15)

Symmetry codes: (iv) *x*−1/4, −*y*+1/4, *z*−1/4.
